# Increased Nuclear Transporter KPNA2 Contributes to Tumor Immune Evasion by Enhancing PD-L1 Expression in PDAC

**DOI:** 10.1155/2021/6694392

**Published:** 2021-03-01

**Authors:** Kai-Xia Zhou, Shan Huang, Li-Peng Hu, Xue-Li Zhang, Wei-Ting Qin, Yan-Li Zhang, Lin-Li Yao, Yanqiu Yu, Yao-Qi Zhou, Lei Zhu, Jianguang Ji, Zhi-Gang Zhang

**Affiliations:** ^1^State Key Laboratory of Oncogenes and Related Genes, Shanghai Cancer Institute, Renji Hospital, School of Medicine, Shanghai Jiao Tong University, Shanghai 200240, China; ^2^Department of Pathophysiology, College of Basic Medical Sciences, China Medical University, Shenyang 110122, China; ^3^Shenyang Engineering Technology R&D Center of Cell Therapy CO. LTD, Shenyang 110169, China; ^4^Clinical Research Centre, Skåne University Hospital, Lund University, Malmö 205 02, Sweden

## Abstract

Pancreatic ductal adenocarcinoma (PDAC) is one of the deadliest malignancies and is known for its high resistance and low response to treatment. Tumor immune evasion is a major stumbling block in designing effective anticancer therapeutic strategies. Karyopherin alpha 2 (KPNA2), a member of the nuclear transporter family, is elevated in multiple human cancers and accelerates carcinogenesis. However, the specific role of KPNA2 in PDAC remains unclear. In this study, we found that expression of KPNA2 was significantly upregulated in PDAC compared to adjacent nontumor tissue and its high expression was correlated with poor survival outcome by analyzing the GEO datasets. Similar KPNA2 expression pattern was also found in both human patient samples and KPC mouse models through IHC staining. Although KPNA2 knockdown failed to impair the vitality and migration ability of PDAC cells *in vitro*, the *in vivo* tumor growth was significantly impeded and the expression of immune checkpoint ligand PD-L1 was reduced by silencing KPNA2. Furthermore, we uncovered that KPNA2 modulated the expression of PD-L1 by mediating nuclear translocation of STAT3. Collectively, our data suggested that KPNA2 has the potential to serve as a promising biomarker for diagnosis in PDAC.

## 1. Introduction

Pancreatic ductal adenocarcinoma (PDAC) is among the most metastatic and lethal cancers in humans; once diagnosed, it is usually in the advanced stage, and the 5-year survival rate is about 9% [1]. To find clues useful for early detection and effective therapy for PDAC, there is much interest in tumor histopathology and molecular characteristics of tumor cells. However, the tumor is a complex disease whose outcome depends largely on the communication between the tumor and its microenvironment. The suppressive tumor microenvironment induced by interactions between cancer cells and stromal cells is critical for PDAC progression and has been implicated in the failure of radiation therapy, chemotherapy, and immunotherapy [2]. Decoding cross-talk between the tumor and its immune contexture is of great importance for exploiting therapy.

The term “immunosurveillance” refers to the physiological process by which the immune system recognizes and kills aberrant cells [3]. Some tumor cells have developed multiple strategies to avoid recognition and elimination by the host immune cells, allowing them to evade immune attack (“immune evasion”) and continue cancer progression [4]. Especially, with immune-privileged nature, PDAC starting from the early preneoplastic state appears to escape from the antitumor immune response [5]. Several molecular mechanisms are involved in PDAC immune evasion. In other words, tumor cells and other cells from the tumor microenvironment can promote an immune privilege status by releasing immunosuppressive cytokines or modulating the expression of immunoregulatory molecules to induce T cell anergy or tolerance, such as the immune checkpoint molecules of B7 family, and LAG3 [6, 7]. One of the most remarkable molecules used by tumor cells to engage T cell immune checkpoints is programmed death ligand 1 (PD-L1). Mechanically, upregulated PD-L1 on the surface of tumor cells can bind to the programmed cell death protein 1 (PD1) which is presented on tumor-infiltrating lymphocytes and subsequently cause blockade of T cell activation and protect tumor cells from T cell-mediated killing [8].

Nucleocytoplasmic transport plays an irreplaceable role in extensive cellular processes, such as gene expression, cell cycle progression, and signal transduction [9]. Dysfunction of nucleocytoplasmic transport is generally observed in many malignant biological behaviors. Karyopherin alpha 2 (KPNA2), which is also known as importin-*α*1 in humans, is one of the seven members of karyopherin *α* family and functions as an adaptor protein for nuclear receptor importin *β* transportation between the nucleus and cytoplasm [10, 11]. In detail, importin *α* serves as an adaptor that links the nuclear localization signal- (NLS-) containing cargo proteins to the nuclear pore complexes (NPCs). When the NLS is recognized, importin *β* docks the ternary complex at the NPC and boosts the translocation of the cargo proteins into the nucleus. During the last decades, many studies have demonstrated that KPNA2 acts as a key oncogenic factor in multiple human cancers including breast cancer, melanoma, liver cancer, and lung cancer [12]. It was upregulated and could transport more transcriptional factors into the nucleus where they modulate gene expression and induce cancer cell growth and invasion. Moreover, it has been reported that KPNA2 was overexpressed in PDAC, but the exact function of KPNA2 in PDAC remains enigmatic [13].

In this study, we figured out the relationship between the KPNA2 expression and PDAC. We identified that KPNA2 is upregulated in PDAC and is associated with poor prognosis. High expression of KPNA2 maintains the expression of PD-L1 by mediating the nuclear translocation of STAT3 and the transcriptional factor of PD-L1. Therefore, upregulated PD-L1 protects PDAC cells from T cells' attack and subsequently proceeds tumor progression. In brief, our findings unveiled a critical role of KPNA2 in tumor immune evasion, which indicated that KPNA2 has the potential to serve as a promising biomarker for PDAC patients.

## 2. Materials and Methods

### 2.1. Data Mining

Five GEO datasets (GSE15471, GSE16515, GSE62452, GSE28735, and GSE71729) and TCGA were used to analyze the KPNA2 expression pattern and detect the prognostic value of KPNA2 in PDAC. The primary data for TCGA datasets were downloaded from https://www.cancer.gov/. The primary data for GEO datasets were downloaded from https://www.ncbi.nlm.nih.gov/geo.

### 2.2. Animal Study

All animal experiments followed the National Institute of Health guidelines for the Care and Use of Laboratory Animals and were approved by the Institutional Animal Care and Use Committee of East China Normal University. As for the orthotopic model, KPC1199-luc cells (1 × 10^5^) were injected in the pancreas of C57BL/6N mice. At the end of the experiment, mice in the various groups were sacrificed and their pancreases were harvested for specific purposes.

### 2.3. Cell Culture and Cell Transduction

Human PDAC cell lines (AsPC-1, Capan1, CFPAC-1, MIA PaCa-2, PANC-1, and PATU8988) and normal HPNE were gained from the Cell Bank of the Chinese Academy of Sciences (Shanghai, China). Mouse PDAC cell line KPC1199-luc was appropriately maintained at Shanghai Cancer Institute, Ren Ji Hospital, School of Medicine, Shanghai Jiao Tong University. All cells were cultured in Roswell Park Memorial Institute (RPMI) 1640 medium or Dulbecco's modified Eagle's medium (DMEM) supplemented with 10% fetal bovine serum and 1% antibiotics (Gibco, 15240062). All cultures were maintained in an incubator at 37°C, 5% CO_2_, and saturated humidity. MIA PaCa-2 and PANC-1 cells were transfected with lentivirus containing a short hairpin RNA (shRNA) sequence against KPNA2 (*Homo sapiens*) or empty vehicle control. KPC1199-luc cells were transfected with lentivirus containing shRNA sequence against KPNA2 (*Mus musculus*) or empty vehicle control. All transfection was conducted in the presence of 6 *μ*g/ml polybrene (Sigma, H9268), and then, cells were screened under 2.5 *μ*g/ml puromycin (Gibco, A1113802) for 14 days. The expression of KPNA2 was detected by qPCR and western blot. The sequence of KPNA2 (*Homo sapiens*) shRNA are 5′- GCTGGTTTGATTCCGAAAT -3′ and 5′- GTGGCTACTTACGTAATCT -3′. The sequence of KPNA2 (*Mus musculus*) shRNA are 5′- GCAGATTCTTCCTACGTTAGT -3′ and 5′- GGTTATTCTTGACGCCATTTC -3′.

### 2.4. Immunohistochemical (IHC)

The protocol of this assay and quantification of the specific protein expression level were performed according to previously reported literature [14]. Primary antibodies used are as follows: KPNA2 (Abcam, ab84440), p-STAT3 (CST, 9145S), CD8 (Abcam, ab217344), and Granzyme B (Immunoway, YT6137).

### 2.5. Proliferation Assay

Cell viability was measured according to the manufacturer's instructions of Cell Counting Kit-8 (SB-CCK8S, Share-Bio, China). Cells with indicated treatment were grown in 96-well plates at 2,500 cells per well and cultured for 24, 48, 72, and 96 hours. At the indicated time point, the culture medium was removed and 10% (volume/volume) CCK-8 was added to each well. After one-hour incubation, the optical density was measured at 450 nm using a microplate reader (M1000 PRO, Tecan). The experiments were performed in a quintuple manner and repeated twice.

### 2.6. Wound Healing Assay

Wound healing assay was conducted using an ibidi culture insert (ibidi, Germany). Cells were seeded into culture insert placed in a 6-well plate. The culture insert was removed while the cells were attached to the dish and enough culture medium was added. Photos were captured at 0 and 24 hours.

### 2.7. Cellular Immunofluorescence

Cells were fixed with 4% paraformaldehyde (Profession and creation, PCDBE008) for 15 min and permeabilized with 0.1% Triton 100 (Beyotime, ST795) for 10 min. Then, cells were blocked with 10% BSA (Biofroxx, 4240GR250) at room temperature for 1 h, incubated with primary antibody against KPNA2 (ab84440, Abcam), and STAT3 (60199-I-Ig, Proteintech) and then with secondary antibodies conjugated with Alexa Fluor-594 (F300605, Abways) or Fluor-488 (A0010031, YEASEN). Antifade Mounting Medium with DAPI (H-1200-10, VECTASHIELD) was used to stain the nucleus. Immunofluorescence images were acquired with a confocal microscope (Leica).

### 2.8. Flow Cytometry

Cells were digested using trypsin (Gibco, 25200056), washed with PBS (Hyclone, SH30256), and suspended in PBS. Tumor tissues from mice were carefully minced and digested using collagenase type IV (Gibco, 2049898) to get single tumor cells. Then, tumor cells were filtered with 70 *μ*m Nylon mesh. After 600 × g centrifugation for 5 min, cells were resuspended in PBS. The cell suspension was incubated with APC anti-mouse CD274 (Biolegend, 124311) for 30 mins at 4°C. Flow cytometry analyses were performed on BD FACSCalibur (BD, USA).

### 2.9. Real-Time PCR

Total RNA was extracted using RNAiso Plus (Takara, 9109) according to the manufacturer's instructions. cDNA was synthesized using the PrimeScript™ RT Master Mix (Takara, RR036A). The RT-PCR was performed using SYBR Green Master Mix (Yeasen, 11202ES03). The 2^−*△△*CT^ method was used to analyze the data, and 18S or *β*-actin was used as a loading control. The primer sequences are listed in Supplementary table 1.

### 2.10. Western Blots

Cells were washed and lysed with RIPA buffer (NCM, WB3100) containing protease inhibitor cocktail (Bimake, B14001) on ice for 10 min. Then, protein lysate followed centrifugation in 4°C for 10 min and the supernatant was collected. Protein supernatants were prepared with 5× SDS loading buffer (Solarbio, P1040) and denatured at 100°C for 5 min. Appropriate protein samples were separated by 4-20% Genshare PAGE gel electrophoresis and electroblotted onto NC membranes using eBlot™ L1 Protein Transfer System (GenScript). The membranes were incubated in 5% nonfat powdered milk (Aangon Biotech, F704BA0001) in TBST (TBS with 0.1% Tween 20) for 1 hour at room temperature, followed by incubation with primary antibodies against specific proteins overnight: *β*-actin (Yeasen, 30101ES50), KPNA2(Abcam, ab84440), and p-STAT3(CST, 9145S). The primary antibodies were diluted in universal antibody diluent (WB500D, NCM). The membranes were washed thrice of 10 min each time and incubated with HPR-conjugated goat anti-mouse (Jackson ImmunoResearch, 115-035-003) or rabbit secondary antibodies (Jackson ImmunoResearch, 111-035-003) for 1 hour at room temperature.

### 2.11. Statistical Analysis

Data were presented as means ± SD or as boxplots, and all statistics were conducted using GraphPad Prism 7.0 and Excel. Statistical analysis was performed using one-way ANOVA, two-way ANOVA, or unpaired Student's *t*-test as appropriate for the dataset. Kaplan-Meier method was used to illustrate the overall survival in patients with PDAC, and significance was determined by log-rank Mantel–Cox test. Functional data are representative of at least triplicates unless otherwise specified. Statistical significance is displayed as ^∗^*P* < 0.05, ^∗∗^*P* < 0.01, ^∗∗∗^*P* < 0.001, and ^∗∗∗∗^*P* < 0.0001; ns: not significant.

## 3. Results

### 3.1. Increased KPNA2 Predicated Poor Prognosis in PDAC

**Figure 1:**
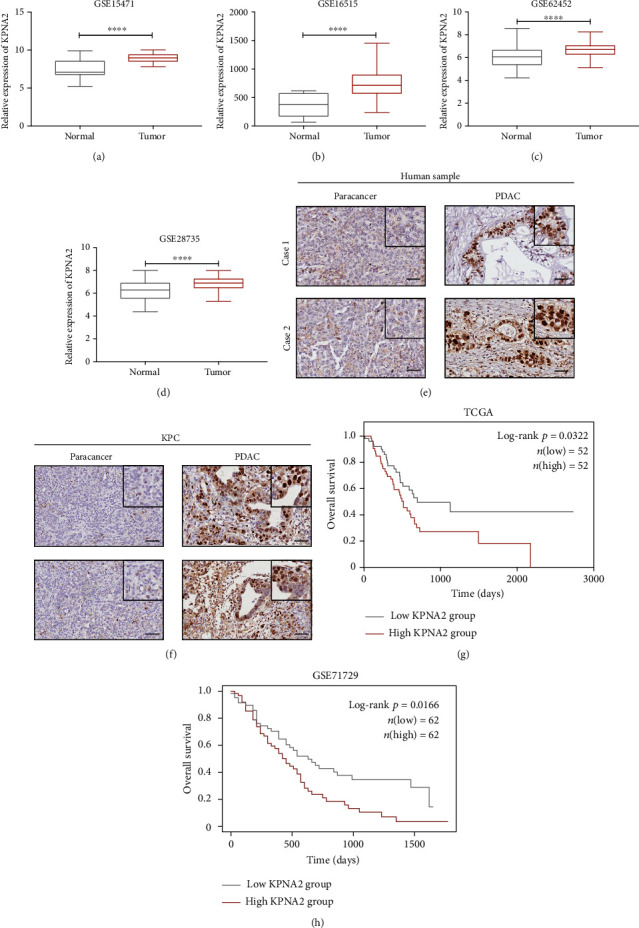
Expression pattern of KPNA2 in PDAC. (a–d) Expression analysis of KPNA2 in tumors and corresponding adjacent nontumor tissues using four independent GEO datasets (GSE15471, GSE16515, GSE62452, and GSE28735) (Student's *t*-test, ^∗∗∗∗^*P* < 0.0001). (e) IHC staining of KPNA2 expression in PDAC tumor (scale bar: 100 *μ*m). (f) IHC staining of KPNA2 expression in the engineered mouse model of KPC (scale bar: 100 *μ*m). (g) Kaplan-Meier overall survival (OS) curves in the TCGA dataset of PDAC patients according to the mRNA expression of KPNA2, the lower quartile value of expression was utilized as a cut-off (log-rank test, *P* = 0.0322). (h) Kaplan-Meier OS curve for the MPC1 expression in the GSE71729 dataset (log-rank test, *P* = 0.0166).

It has been previously reported that dysfunction of nucleocytoplasmic transport is related to the progression of multiple human cancers. In this study, we compared the expression of nuclear import adaptors between the GTEx dataset and PDAC samples in the TCGA dataset to determine the potential maladjustment genes involved in tumor progression. As shown in supplementary figure 1 (a), KPNA7 had the greatest log2 (fold change) and KPNA2 was running behind. KPNA7 was reported to promote malignant properties of pancreatic cancer cells *in vitro*, while the exact function of KPNA2 in PDAC remains unclear. To explore the clinical significance of KPNA2, we downloaded four independent GEO datasets of pancreatic cancer, including GSE15471, GSE16515, GSE62452, and GSE28735. Further analysis showed that the mRNA expression level of KPNA2 was significantly increased in tumor specimens in comparison with that in the adjacent nontumor tissues (Figures 1(a)–1(d)). To validate the clinical relevance of KPNA2 in PDAC, we examined the expression pattern of KPNA2 in mouse and human PDAC tissues. Higher protein level of KPNA2 clearly existed in the PDAC tissues compared with the adjacent nontumor tissues derived from PDAC patients and the engineered mouse models of LSL-**K**ras^G12D/+^, LSL-Tr**p**53^R172H/+^, and Pdx1-**C**re (KPC) (Figures 1(e) and 1(f)). Additionally, we observed that KPNA2 appeared to be predominantly situated in the nucleus of cancer cells. Furthermore, high expression of KPNA2 in cancer tissues was significantly associated with a poor prognosis in patients with PDAC (Figures 1(g) and (h)).

### 3.2. KPNA2 Knockdown Inhibited the Growth of PDAC Cells *In Vivo* Rather than *In Vitro*

**Figure 2:**
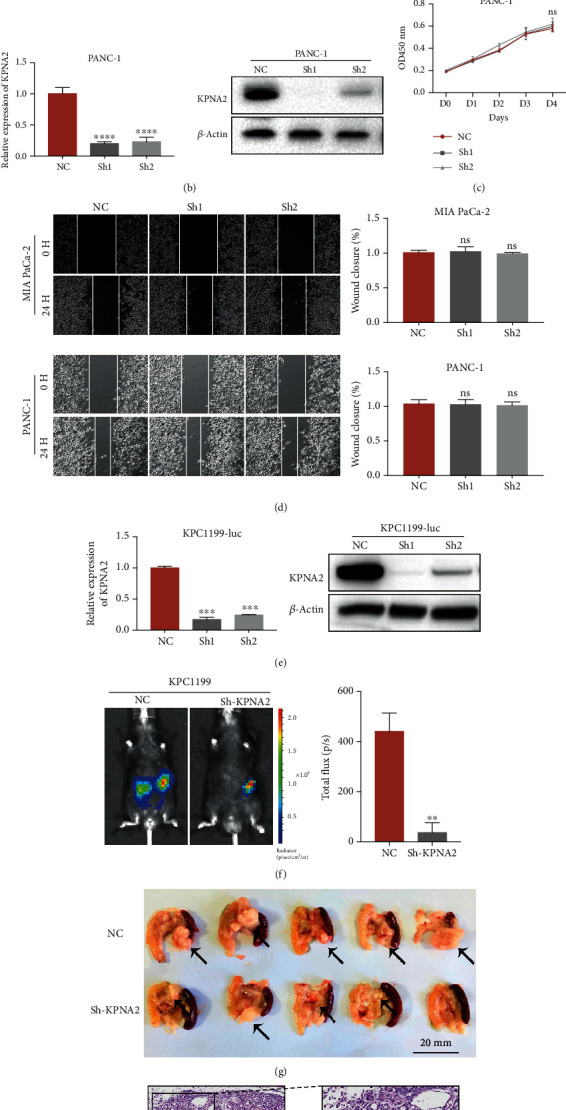
KPNA2 knockdown inhibited the growth of PDAC cells *in vivo* rather than *in vitro*. (a, b) KPNA2 silencing by sh-RNA-KPNA2 in MIA PaCa-2 and PANC-1 cells. (c) CCK8 assays of the control group and KPNA2 knockdown group of MIA PaCa-2 and PANC-1 cell lines. (d) Wound healing assays of the control group and KPNA2 knockdown group of MIA PaCa-2 and PANC-1 cell lines. (e) KPNA2 silencing by sh-RNA-KPNA2 in KPC1199-luc cell. (f) Bioluminescence imaging and luminescence intensity of orthotopic tumor growth. (g) Pancreas of C57BL/6N mice orthotopically transplanted stable KPNA2-knockdown KPC1199-luc cells and control cells. (h) Representative images of H&E staining of orthotopic PDAC model mice at 21 days.

To evaluate the role of KPNA2 on the vitality and motility of PDAC cells, the CCK-8 assay and wound healing assay were performed. Firstly, we examined the mRNA and protein levels of KPNA2 in human PDAC cell lines. As shown in supplementary figure 1 (b) and (c), the mRNA and protein expressions of KPNA2 in PDAC cell lines MIA PaCa-2 and PANC-1 are relatively higher. Next, stable KPNA2-knockdown MIA PaCa-2 and PANC-1 cell lines were established by infecting with lentivirus carrying the KPNA2-shRNA (Figures 2(a) and 2(b)). Intriguingly, KPNA2 knockdown failed to impair the vitality and migration ability in both MIA PaCa-2 and PANC-1 cells (Figures 2(c) and 2(d)). To verify whether KPNA2 modulates PDAC progression *in vivo*, an orthotopic xenograft model was generated. Firstly, the luciferase-expressing KPC1199 cells (a mouse PDAC cell line derived from KPC mouse) was infected with lentivirus carrying the KPNA2-shRNA to establish stable KPNA2-knockdown cells. Then, the knockdown efficiency was tested by qPCR as well as western blot assays (Figure 2(e)). The orthotopic PDAC model was established via orthotopically transplanting stable KPNA2-knockdown KPC1199-luc cells. As predicted, knockdown of KPNA2 significantly reduced the tumor burden in the orthotopic xenograft model by using the in vivo imaging system (Figure 2(f)). Moreover, the number as well as size of tumor lesions in the KPNA2 silencing group was smaller than that in the control group (Figure 2(g)). Histological examination indicated that KPNA2 knockdown decreased the tumorigenicity ability of PDAC *in vivo* (Figure 2(h)).

### 3.3. Increased KPNA2 Promoted the PD-L1 Expression and Thereby Contributed to Tumor Immune Evasion

**Figure 3:**
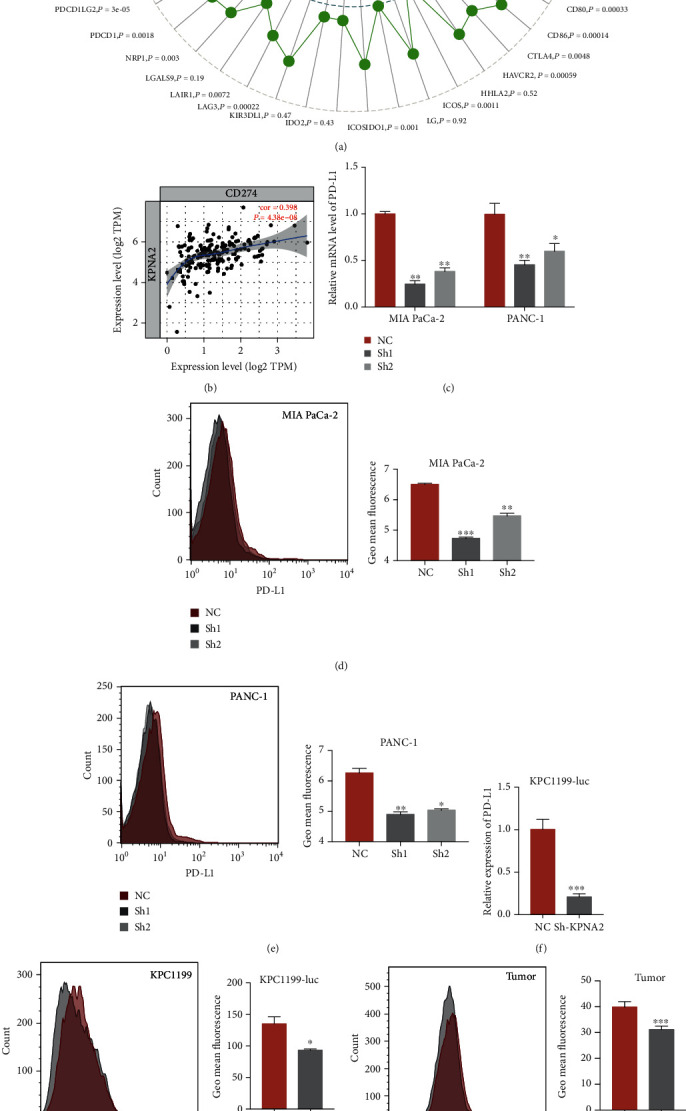
Increased KPNA2 promoted PD-L1 expression and thereby contributed to tumor immune evasion. (a) Gene-immune analysis of KPNA2 in PDAC conducted on Sanger box. (b) The correlation between expression levels of KPNA2 and PD-L1 in PDAC. (c) Relative mRNA expression level of PD-L1 target genes in MIA PaCa-2 and PANC-1 cells with sh-KPNA2 or control vector. (d, e) Protein level of PD-L1 on the surface of MIA PaCa-2 and PANC-1 cells with sh-KPNA2 or control vector. (f, g) Relative mRNA expression level and protein level of PD-L1 in KPC1199-luc cells with sh-KPNA2 or control vector. (h) Protein level of PD-L1 on the surface of tumor cells derived from orthotopic PDAC model mice orthotopically transplanted KPC1199-luc cells with sh-KPNA2 or control vector. (i) CD8 infiltration and granzyme B expression in mouse tumor tissue detected by IHC (scale bar: 100 *μ*m).

Since silencing KPNA2 significantly impeded the *in vivo* tumor growth but failed to impair the vitality and migration ability of PDAC cells *in vitro*, we guessed whether upregulated KPNA2 leads to tumor progression via contributing to tumor immune evasion. To verify this hypothesis, we conducted Gene-Immune Analysis using Sanger box (http://sangerbox.com/Index) and TIMER: tumor immune estimation resource (https://cistrome.shinyapps.io/timer/). As expected, the results revealed that the expression of CD274, which is also known as PD-L1, is highly correlated with KPNA2 expression (Figures 3(a) and 3(b)). Furthermore, KPNA2 silencing MIA PaCa-2 and PANC-1 cells exhibited significantly reduced PD-L1 expression than the control cells (Figures 3(c)–3(e)). Consistent with the results in human cells, genetic inhibition of KPNA2 impaired the expression of PD-L1 in KPC1199-luc cells (Figures 3(f) and 3(g)). To determine the expression of PD-L1 in tumor lesions, we minced and digested the tumors into single cells and stained it by PD-L1 antibody. FCM results showed that the KPNA2 knockdown group exhibited reduced PD-L1 expression than the control group (Figure 3(h)). Moreover, as shown in Figure 3(i), the control group specimens represented less CD8+ T cell infiltration and granzyme B expression in the tumor stroma while compared with KPNA2 knockdown group (Figure 3(i)).

### 3.4. KPNA2 Maintained the Expression of PD-L1 by Mediating Nuclear Translocation of STAT3

**Figure 4:**
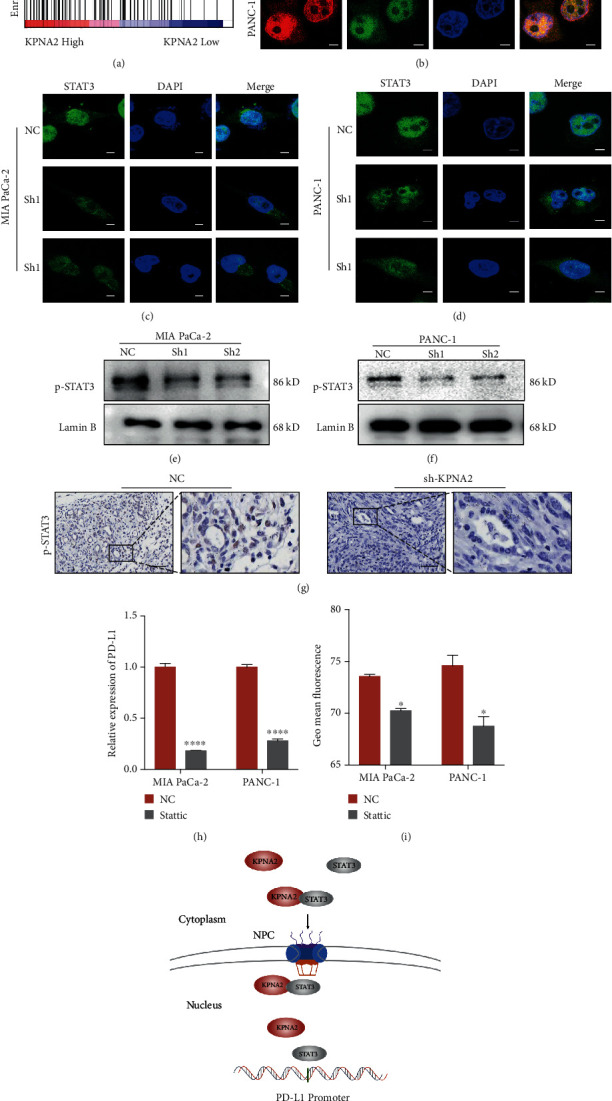
KPNA2 maintained the expression of PD-L1 by mediating nuclear translocation of STAT3. (a) GSEA analysis of KPNA2 expression in PDAC using the TCGA dataset. NES: normalized enrichment score. (b) Coimmunofluorescence of KPNA2 and STAT3 in MIA PaCa-2 and PANC-1 cells (scale bar: 10 *μ*m). (c, d) KPNA2 knockdown could inhibit the nuclear translocation of STAT3 in MIA PaCa-2 and PANC-1 cells (scale bar: 10 *μ*m). (e, f) The expression of nuclear p-STAT3 was detected in control and KPNA2 knockdown PDAC cells. Lamin B was used as the loading control of nuclear protein. (g) IHC staining of p-STAT3 in mouse tumor lesions inoculated with KPC1199-luc cell treatment with sh-KPNA2 or control vector (scale bar: 100 *μ*m). (h, i) Relative mRNA expression level and protein level of PD-L1 in PDAC cells treated with 2.5 *μ*M stattic, STAT3 inhibitor. (j) Proposed model for nuclear transporter KPNA2 promotes the PDAC progress.

To gain comprehensive insight into how KPNA2 promotes PD-L1 expression, the TCGA database was used to perform gene set enrichment analysis (GSEA). The results indicated that KPNA2 alteration of the IL-6-JAK-STAT3 pathway was correlated with KPNA2 expression when the mRNA expression quartile was set as a cut-off (Figure 4(a)). Previous studies have reported that STAT3 directly binds to the promoter of PD-L1, which increases subsequent PD-L1 expression [15, 16]. In view of the fact that KPNA2 mainly functions as a nuclear transport adaptor, we investigated whether KPNA2 helps the nuclear translocation of STAT3 through immunofluorescence staining of KPNA2 and STAT3 in MIA PaCa-2 and PANC-1 cells. As shown in Figure 4(b), STAT3 surely colocalized with KPNA2 in cell nucleus. And silencing KPNA2 weakened the nuclear distribution of STAT3 in both MIA PaCa-2 and PANC-1 cells when compared to control cells (Figures 4(c) and 3(d)). Consistently, obviously decreased distribution of nuclear p-STAT3, activated STAT3, was presented in KPNA2-knockdown cells (Figures 4(e) and 3(f)). Moreover, as shown in Figure 4(g), the control group mouse specimen represented more p-STAT3 in nuclear while compared with the KPNA2 knockdown group. The expression of PD-L1 was inhibited by the STAT3 inhibitor stattic as determined by qPCR and FCM (Figures 3(g) and 3(h)). Together, these data suggested that KPNA2 mediates the nuclear translocation of STAT3, which subsequently maintains the expression of PD-L1.

## 4. Discussion

The immune system has the potential to recognize and eliminate tumor cells, and many types of human tumors can escape immune surveillance to enhance their survival [17–19]. Deciphering specific biological processes involved may necessitate interpreting the responses of tumors to immunotherapy and provide strategies for overcoming resistance therapeutically. This study demonstrated that KPNA2 expression was upregulated and predicted poor prognosis in PDAC. Through conducting functional and mechanistic studies, we identified that KPNA2, a central importin in nuclear import, maintained PD-L1 expression by mediating nuclear translocation of STAT3 and ultimately led to tumor immune evasion (Figure 4(j)).

Nucleocytoplasmic transport is a highly coordinated process involving numerous proteins as well as large complexes working in concert at the nuclear envelope. This process delicately balances cell growth and death mechanisms in cells. Dysregulation of this fundamental process may affect many other important cellular processes such as tumor growth, inflammatory response, cell cycle, and apoptosis. As reviewed previously, KPNA2 has been proposed to act as a diagnostic, prognostic, or predictive marker for various malignancies, including lung, gastric, bladder, breast, brain, prostate, ovarian, and esophageal cancers [20]. In terms of functional data, KPNA2 overexpression induced cell migration, cell viability, and cell proliferation of cancer cells, which indicates that abnormal expression of nuclear transporters in cancer cells interferes with cellular homeostasis and thereby contributes to tumor pathogenesis. Previous study of the adenoma carcinoma sequence indicated that KPNA2 expression is associated with carcinogenesis of IPMN [21]. Additionally, it was reported that primary pancreatic intraepithelial neoplasia with high expression of KPNA2 emerged in aged cysteine414-alanine mutant mCRY1 transgenic mice [22]. However, the underlying mechanisms are vague. In this study, we verified that aberrantly increased KPNA2 contributed to PDAC progress by maintaining tumor immune evasion rather than impacting the malignant properties of tumor cells. Laurila et al. demonstrated that silencing KPNA7, the nearest relative of KPNA2, inhibits the malignant properties of pancreatic cancer cells *in vitro* [23]. It might be the reason genetic inhibition of KPNA2 has no effect on the viability and migration of pancreatic cancer cells *in vitro*. In another word, KPNA7 may pay “compensation.” To sum up, we elucidated that KPNA2 is involved in immune privilege of tumorigenesis.

The immune system interacts intimately with tumors during disease development and progression to metastasis [24]. Immune evasion is one of the prominently recognized hallmarks of cancer. Mechanisms leading to an evasion of immune attack include the selection of tumor variants to immune effectors and progressive formation of an immune suppressive environment within the tumor [25]. Immunity in PDAC patients is attenuated and the associated immune evasion is an underinvestigated field. Over the entire process of disease development, the heterogeneity of cancer cells gradually expands. Previous studies have indicated that membranous PD-L1 expression is scarce in PDAC and PD-L1 positivity rate is low in PDAC when evaluated using a companion diagnostic assay [26]. However, it is noted that PD-L1 expression in PDAC is a poor prognostic factor in patients with high CD8+ tumor-infiltrating lymphocytes [27]. PD-L1 expression could be activated in tumor cells either by oncogenic signaling or by inflammatory cytokines, particularly interferon gamma, as a result of the adaptive immune response. A succession of studies demonstrated that oncogenic signaling would activate PD-L1 expression in PDACs [28–30]. For the development of more practical immunotherapies, it is first necessary to clarify the immunological escape mechanisms. In this study, we described that nucleocytoplasmic transport of STAT3 participates in PD-L1 expression, which provides a perspective that nucleocytoplasmic transport makes a contribution to the regulation of PD-L1 expression.

Nuclear transport protein KPNA2 combines with cargo proteins with NLS and thus regulates the nuclear translocation of various proteins, including many different transcription factors. Among these proteins, most of them are mainly involved in participating in PI3K/AKT, MAPK, Wnt/*β*-catenin, and EMT-related pathways to affect the growth, invasion, and metastasis of tumors. In detail, it has been reported that the overexpression level of KPNA2 and its cargo protein OCT4 in bladder cancer tissues were significantly related with poor prognosis of patients [31]. Our data confirmed that KPNA2 mediates the STAT3 nuclear import. Many more studies focused on nucleocytoplasmic transport of immunoregulatory elements should be conducted in the future.

## 5. Conclusions

In conclusion, we demonstrated that increased nuclear transporter KPNA2 was closely correlated with PDAC patient's immune evasion, and thereby led to a poor outcome. Moreover, KPNA2-driven nuclear translocation of STAT3 contributed to PD-L1 upregulation in PDAC cells. This means that KPNA2 has the potential to be a diagnostic biomarker for PDAC patients.

## Data Availability

The data used to support the findings of this study are available from the corresponding author upon request.

## Supplementary Materials

Supplementary materials “See Figure S1 and Table S1 in the Supplementary Material for the expression analysis of KPNA2 in PDAC cell lines and primer sequences respectively.”
