# Supplementary material for: Increased Nuclear Transporter KPNA2 Contributes to Tumor Immune Evasion by Enhancing PD-L1 Expression in PDAC
Source: J Immunol Res. 2021 Mar 1;2021:6694392. doi: 10.1155/2021/6694392 (PMC7939744; doi:10.1155/2021/6694392)
Supplement: Supplementary materials — “See Figure S1 and Table S1 in the Supplementary Material for the expression analysis of KPNA2 in PDAC cell lines and primer sequences respectively.” [file 6694392.f1.docx]

**Increased Nuclear Transporter KPNA2 Contributes to Tumour Immune Evasion by Enhancing PD-L1 expression in PDAC**

Kai-Xia Zhou^1^†, Shan Huang^1^†, Li-Peng Hu^1^†, Xue-Li Zhang^1^, Wei-Ting Qin^1^, Yan-Li Zhang^1^, Lin-Li Yao^1^, Yanqiu Yu^3, 4^, Yao-Qi Zhou^1^, Lei Zhu^1*^, Jianguang Ji^2*^, Zhi-Gang Zhang^1*^

^1^ State Key Laboratory of Oncogenes and Related Genes, Shanghai Cancer Institute, Renji Hospital, School of Medicine, Shanghai Jiao Tong University, Shanghai 200240, PR China.
^2^ Clinical Research Centre, Skåne University Hospital, Lund University, Malmö 205 02, Sweden.

^3^ Department of Pathophysiology, College of Basic Medical Sciences, China Medical University, Shenyang 110122, PR China.

^4^ Shenyang Engineering Technology R&D Center of Cell Therapy CO.LTD, Shenyang 110169, PR China.

*Corresponding author.

Address correspondence to: Zhi-Gang, Zhang, State Key Laboratory of Oncogenes and Related Genes, Shanghai Cancer Institute, Ren Ji Hospital, School of Medicine, Shanghai Jiao Tong University, 800 Dongchuan Road, Shanghai 200240, PR China, E-mail: zzhang@shsci.org; or Jianguang, Ji, Clinical Research Centre, Skåne University Hospital, Lund University, Malmö 205 02, Sweden, E-mail: Jianguang.ji@med.lu.se; or Lei Zhu, State Key Laboratory of Oncogenes and Related Genes, Shanghai Cancer Institute, Ren Ji Hospital, School of Medicine, Shanghai Jiao Tong University, 800 Dongchuan Road, Shanghai 200240, PR China, E-mail: lzhu@shsci.org

† These authors contributed equally to this work.

**Supplementary Figure.1**


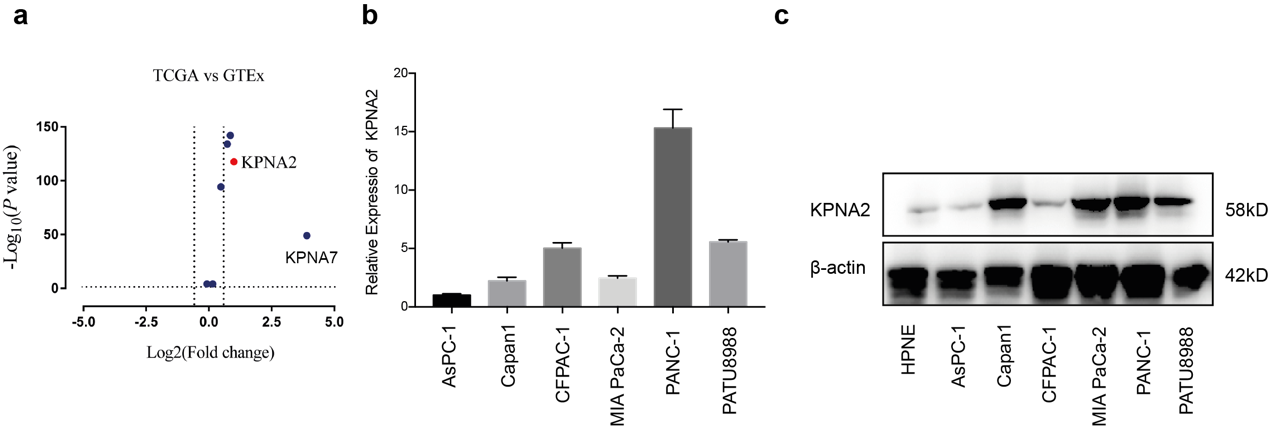


**Supplementary Figure 1.** (a) Volcano plot showed fold changes (x-axis) and corresponding P values (log10, y-axis) of nuclear import adaptor genes between GTEx and PDAC samples in the TCGA dataset (Student’s t-test). (b) Relative mRNA expression of KPNA2 in 6 PDAC cell lines. (c) Relative protein expression of KPNA2 in 6 PDAC cell lines and HPNE cell.

**Supplementary Table.1**

Table 1: Sequences of primers used for real-time PCR.

| Primer | Sequence 5’-3’ |
| --- | --- |
| Hsa-KPNA2 forward | GGCACTGTAAATTGGTCTGTTGA |
| Hsa-KPNA2 reverse | CCTGGCAGCTTGAGTAGCTT |
| Mmu-KPNA2 forward | ATGTCCACGAACGAGAATGCT |
| Mmu-KPNA2 reverse | AAGGAGCTGACGTTTCTTCTTTT |
| Hsa-PD-L1 forward | TGGCATTTGCTGAACGCATTT |
| Hsa-PD-L1 reverse | TGCAGCCAGGTCTAATTGTTTT |
| Mmu-PD-L1 forward | GCTCCAAAGGACTTGTACGTG |
| Mmu-PD-L1 reverse | TGATCTGAAGGGCAGCATTTC |
| Hsa-18S forward | TGCGAGTACTCAACACCAACA |
| Hsa-18S reverse | GCATATCTTCGGCCCACA |
| Mmu-β-actin forward | CTCTGGCTCCTAGCACCATGAAGA |
| Mmu-β-actin reverse | GTAAAACGCAGCTCAGTAACAGTCCG |
